# Assessing the Usage and Usability of a Mental Health Advice Telephone Service in Uganda: Mixed Methods Study

**DOI:** 10.2196/65692

**Published:** 2024-10-21

**Authors:** Johnblack K Kabukye, Juliet Nakku, Jackline Niwemuhwezi, James Nsereko, Rosemary Namagembe, Iris Dorothee Emilie Groen, Ritah Neumbe, Denis Mubiru, Caroline Kisakye, Roseline Nanyonga, Marie Sjölinder, Susanne Nilsson, Caroline Wamala-Larsson

**Affiliations:** 1 Swedish Program for ICT in Developing Regions (SPIDER) Department of Computer and Systems Science Stockholm University Stockholm Sweden; 2 Uganda Cancer Institute Kampala Uganda; 3 Butabika National Referral Mental Hospital Kampala Uganda; 4 Hutchinson Centre Research Institute of Uganda Uganda Cancer Institute Kampala Uganda; 5 MoleMann Mental Health Almere Netherlands; 6 Research Institutes of Sweden (RISE) Stockholm Sweden; 7 Unit for Integrated Product Development and Design Department of Machine Design KTH Royal Institute of Technology Stockholm Sweden

**Keywords:** mHealth, mental health, telephone service, usability, satisfaction, evaluation, mixed method, Uganda, Africa

## Abstract

**Background:**

Harnessing mobile health (mHealth) solutions could improve the delivery of mental health services and mitigate their impact in Uganda and similar low-resource settings. However, successful adoption requires that mHealth solutions have good usability. We have previously implemented a telephone service to provide mental health information and advice in English and Luganda, utilizing an automated interactive voice response (IVR) system linked to live agents, including mental health care workers and peer support workers.

**Objective:**

This study aims to assess the usage and usability of this mental health telephone service.

**Methods:**

We obtained usage data from the system’s call logs over 18 months to study call volumes and trends. We then surveyed callers to gather their characteristics and assess usability using the Telehealth Usability Questionnaire. Additionally, call recordings were evaluated for conversation quality by 3 independent health care professionals, using the Telephone Nursing Dialogue Process, and correlations between quality and usability aspects were investigated.

**Results:**

Over 18 months, the system received 2863 meaningful calls (ie, calls that went past the welcome message) from 1125 unique telephone numbers. Of these, 1153 calls (40.27%) stopped at the prerecorded IVR information, while 1710 calls (59.73%) opted to speak to an agent. Among those who chose to speak with an agent, 1292 calls (75.56%) were answered, 393 calls (22.98%) went to voicemail and were returned in the following working days, and 25 calls (1.46%) were not answered. Usage was generally sustained over time, with spikes in call volume corresponding to marketing events. The survey (n=240) revealed that most callers were caregivers of patients with mental health issues (n=144, 60.0%) or members of the general public (n=46, 19.2%), while a few were patients with mental health issues (n=44, 18.3%). Additionally, the majority were male (n=143, 59.6%), spoke English (n=180, 75.0%), had postsecondary education (n=164, 68.3%), lived within 1 hour or less from Butabika Hospital (n=187, 77.9%), and were aged 25-44 years (n=160, 66.7%). The overall usability score for the system was 4.12 on a 5-point scale, significantly higher than the recommended target usability score of 4 (*P*=.006). The mean scores for usability components ranged from 3.66 for reliability to 4.41 for ease of use, with all components, except reliability, scoring higher than 4 or falling within its CI. Usability scores were higher for Luganda speakers compared with English speakers, but there was no association with other participant characteristics such as sex, distance from the hospital, age, marital status, duration of symptoms, or treatment status. The quality of call conversations (n=50) was rated at 4.35 out of 5 and showed a significant correlation with usability (Pearson r=0.34, *P*=.02).

**Conclusions:**

We found sustained usage of the mental health telephone service, along with a positive user experience and high satisfaction across various user characteristics. mHealth solutions like this should be embraced and replicated to enhance the delivery of health services in Uganda and similar low-resource settings.

## Introduction

Mobile health (mHealth) solutions have been widely implemented in Africa to support various clinical goals and processes, including medication adherence, appointment attendance, patient communication, health education, clinical data collection and reporting, and clinical decision support, and guideline adherence [1-7]. Generally, positive results have been reported regarding acceptability, usability, and clinical outcomes; however, most implementations have focused on HIV/AIDS and maternal and child health, with limited application in other noncommunicable diseases such as cancer, hypertension, and diabetes [1-6]. Studies on the implementation of mHealth in mental health in Africa are limited. For instance, in a meta-analysis of mobile phone–based interventions for mental health conducted by Goldberg et al [8], none of the 145 identified randomized trials originated from Africa. Another review by Ding et al [9], which focused on mHealth for mental health among youth, found that only 3 out of 151 studies were from Africa (Nigeria). Similarly, in a review of mHealth in Sub-Saharan Africa by Aboye et al [1], only 2 out of 59 studies addressed mental health conditions: an app for dementia screening by lay health workers in Tanzania and a case study of telepsychiatry in Kenya following the outbreak of COVID-19. Other reviews by Ødegård et al [4] (31 clinical trials on 2-way SMS text message interventions) and Osei et al [5] (12 studies on mHealth for disease diagnosis and treatment in Africa) did not include any studies focused on mental health.

A key issue in implementing mHealth solutions is usability. The International Organization for Standardization [10] defines usability as the degree to which a product can be used by specific users to achieve particular goals with effectiveness, efficiency, and satisfaction in a given context. Usability directly affects users’ intention to adopt and continue using technology [11], and thus, ensuring good usability is crucial for engagement with mental health mHealth solutions [12,13]. Moreover, individuals with mental illnesses, particularly in Africa, often face challenges such as not owning mobile devices or sharing them with family members or caregivers, which compromises privacy and confidentiality. Many live in rural areas with poor telecommunication coverage and limited access to electricity for charging devices, have low general and digital literacy, and experience significant stigma, among other difficulties [14-16]. These factors can hinder their ability to learn and use mHealth solutions or affect their satisfaction with them [12,13]. Mental health mHealth solutions, particularly smartphone apps, have also been criticized for being unnecessarily complex [13] and for imposing a high personal quantification burden [12], as they often require users to frequently input data about their behavior or mood for ecological momentary assessments.

Therefore, conducting research on the usability of mental health mHealth interventions [17] is crucial to inform improvements in their design, implementation strategies, and the evidence base for these technologies in Africa. Additionally, because usability is subjective, studying actual usage (eg, the number of returning users or the type of information sought) is essential, as it provides deeper insights into user satisfaction, perceived utility, and their unique challenges and needs.

Uganda, like many other low- and middle-income countries, faces a significant mental health burden and a substantial gap in mental health care. Approximately 1 in 3 Ugandans experience some form of mental illness, with depression (22.2%) and anxiety (20.2%) being the most prevalent [18,19]. Additionally, Uganda has one of the highest per-capita alcohol consumption rates, with alcohol and substance use disorders being widespread, particularly among men, while also affecting children and youth [20]. Several factors contribute to this critical situation, including a severe shortage of mental health care workers and underfunded and limited mental health facilities that are often distant, overcrowded, and prone to medication shortages. Additionally, a lack of awareness and negative sociocultural norms and beliefs hinder health-seeking behavior [21-23]. Previously, we implemented a mental health advice telephone service at the Butabika National Mental Referral Hospital in Kampala, Uganda [24] as a step toward addressing the challenges in Uganda’s mental health care system. This paper evaluates the usage and usability of this tele–mental health service.

## Methods

### Study Design

A detailed description of the development of the mental health service is provided elsewhere [24]. In summary, we used a mixed methods, user-centered, and participatory design approach to assess the information needs and mental health care challenges in Uganda. Based on this assessment, we cocreated and implemented a telephone service that delivers mental health information through an automated interactive voice response (IVR) system, along with the option to connect users to a live agent for personalized advice. The service is available 24/7, accessible via any mobile phone without the need for internet, and is toll-free (reverse-billed). The live agents comprise professional health care workers, including nurses, psychologists, and psychiatric clinical officers, as well as peer support workers—individuals recovering from mental illnesses who share their lived experiences.

This paper assesses the usage and usability of the service through a quantitative study that utilizes telephone system logs to evaluate usage patterns, a cross-sectional telephone survey of callers to assess usability, and rating of call conversation quality by healthcare workers. The study follows the Statement on Reporting of Evaluation Studies in Health Informatics (STARE-HI) guidelines [25].

### Telephone System Usage

#### Data Collection

Data were obtained from the system call logs, which included the phone number, time stamp, duration, and selected IVR menu options. The data covered a period of 18 months, from August 2022, when the system deployment and testing were completed, to February 2024.

#### Analysis

Trends across calendar time, day of the week, and time of day were extracted from the call logs, along with the language used and the destination of the call (IVR, live agent, or voicemail). The data were summarized using descriptive statistics, including frequencies, percentages, medians, and ranges. Graphs were created to visualize these trends.

### Usability Survey

#### Participants

Participants included individuals who had called the telephone system. Survey calls were made once or twice a week, and participants were recruited sequentially from the call logs of the preceding days. A survey log was maintained in a spreadsheet to track callers who had been successfully surveyed versus those for whom the survey could not be completed, such as instances where their phones were unreachable. If more than a month had passed since an individual last called the system without being successfully surveyed, they were excluded from the list of eligible participants, as it was deemed too long for the caller to accurately recall their call experience. The surveys were conducted in either English or Luganda, depending on the participant’s preference. Data were entered in real-time into a Google Form (Google LLC/Alphabet Inc.), which was configured to shuffle the order of the questionnaire items to minimize any potential ordering effects [26].

The survey was administered by trained research assistants, including nurses, peer support workers, and psychiatric clinical officers, who were part of the project team and served as call agents. In the month leading up to data collection, the first author (JKK) trained the research assistants on the questionnaire content, the process for obtaining informed consent over the phone, and techniques to avoid “leading” respondents toward specific answers. Additionally, the first author (JKK) monitored quality control by listening to at least four randomly selected survey calls each month.

#### Sample Size

We estimated the sample size using the recommendation by Park and Jung [27], which suggested a target of 241 participants for a Likert scale encompassing 5 dimensions or usability attributes. This estimation assumes a correlation coefficient of 0.5 and a tolerable error margin of 5%, allowing for a maximum of 4 items per dimension if each dimension is analyzed separately.

#### Data Collection

We utilized the Telehealth Usability Questionnaire (TUQ), developed and validated by Parmanto et al [28]. The TUQ builds on established usability questionnaires and technology acceptance theories, aiming to evaluate a wide range of telehealth setups. Unlike traditional videoconferencing systems designed for single purposes and typically operated by clinicians or technical teams, the TUQ applies to both general-purpose computers and mobile phones. The TUQ can be used to evaluate usability from the patient’s perspective. The original questionnaire comprises 21 items that cover 5 usability attributes: usefulness, ease of use and learnability, interface quality, interaction quality, and reliability, as well as 1 overall satisfaction dimension: satisfaction and future use. For this study, the language was adapted to fit the context by replacing “telehealth system” with “Butabika call center service.” Additionally, the item “the system is simple and easy to understand” was modified to “the information is simple and easy to understand.” The item “I believe I could become productive quickly using this system,” deemed irrelevant to our survey, was also removed. Each item was scored on a 5-point Likert scale, with 1 representing the lowest score and 5 the highest. Additionally, the questionnaire included questions about demographic characteristics and mental health information, as well as a section for free-text comments.

#### Analysis

Usability survey data were analyzed by summarizing participants’ characteristics (frequencies and percentages). Means and SDs were calculated for the TUQ items, subscales (usability attributes), and the overall scale. The scale’s reliability was assessed using Cronbach α. Differences in usability scores among participants’ characteristics were analyzed using the 2-tailed, unpaired *t* test (for language, sex, distance from the hospital, and duration of illness) and ANOVA (for caller category, level of education, and marital status).

The free-text comments were analyzed using a deductive approach [29]. The first (JKK) and fifth (RNamagembe) authors independently coded the comments into themes related to participants’ general opinions about the system, usability challenges, and recommendations for improvement.

### Call Conversation Quality Rating

#### Participants

The quality assessment was conducted by the third, fourth, and fifth co-authors (JaNi, JaNs, RNamagembe), all of whom have been involved in the development and operation of the call center system. JaNi is a social worker, and JaNs is a psychologist at Butabika Hospital, while RNamagembe is a nurse at the Uganda Cancer Institute.

#### Data Collection

Fifty recordings of telephone conversations with the usability survey participants were randomly selected and independently assessed using the telephone nursing dialogue process [30]. The assessment focused on various aspects, including building *rapport*, actively *listening*, gathering and *analyzing* information, informing and *motivating* the caller, and *ending* with mutual agreement and safety netting. The agent who answered the call (peer support work vs professional health care provider) was also recorded, as well as the call issue, that is, if it was solvable on the phone (defined as “caller needed general information such as how to access mental health services, was providing information to us such as reporting a patient who “escaped,” or just checking if the system works”) or not (defined as “a thorough clinical assessment was needed, prescription of medication, urgent intervention for a patient with suicidal ideation, etc”).

The recordings were accessed via a password-protected, time-limited cloud folder, and the assessments were documented in Google Forms.

#### Analysis

The ratings of the call conversations were summarized as means and SDs. The reliability of the quality rating scale was evaluated using Cronbach α. Additionally, the correlation between the ratings provided by health professionals and the callers’ usability scores was computed to determine if a relationship exists. Chi-square tests were conducted to assess associations between the agent type and the call issue.

All data cleaning and analyses of usage, usability, and conversation quality were conducted using MS Excel (version 16; Microsoft Corp.) and SPSS (version 29; IBM Corp.).

### Ethics Considerations

Ethical approval for the research study was obtained from the Makerere University School of Public Health Research Ethics Committee (approval number SPH-2021-153) and the Uganda National Council of Science and Technology (approval number HS1868ES). The research assistant obtained informed consent from the participants before administering the survey, clarifying that participation was voluntary and would not affect their medical care. As compensation for their time, participants received UGX 5000 (approximately US $1.3) in phone credit, as recommended by the research ethics committee.

## Results

### Usage Patterns

From August 2022 to February 2024, a total of 5120 calls were made to the system. Of these, 2257 calls (44.08%) did not interact with the system; these callers were primarily checking to confirm the system’s operation or failed to understand the IVR instructions, resulting in no selection of IVR options. The remaining 2863 calls (55.92% of all calls, from 1125 unique telephone numbers) involved meaningful interactions, where callers chose options from the IVR menu. Notably, nearly half of these calls (1153 calls, or 40.27%, from 609 unique phone numbers) ended after accessing prerecorded IVR information. Out of the total calls (n=2863), 1710 (59.73%) opted to speak with a live agent. Among these, 1292 calls (75.56%, from 593 unique phone numbers) were answered, 25 calls (1.46%, from 25 unique numbers) were not answered, and 393 calls (22.98%, from 254 unique phone numbers) occurred during out-of-office hours and were directed to voicemail; agents returned these calls within the following working days. In total, the calls accumulated to 138.3 hours, with each unique number making a median of 2 calls (minimum=1, maximum=77), and the average duration of each call was approximately 3 minutes. The distribution of calls in English was roughly equal to that in Luganda, and there was no significant difference in call duration (to the agents) between the 2 languages (mean 2.99 vs 3.06 minutes, *P*=.31). Details per call type are presented in Table 1.

**Table 1:** Summary of the calls.

Call type	IVR^a^	Agent	Voicemail	Overall^b^
Number of calls, n	1153	1292	393	2838
Total duration of calls (hours)	49.5	65.0	23.7	138.3
Average duration of each call (minutes)	2.6	3.0	3.6	2.9
Calls in Luganda, n (%)	633 (54.90)	656 (50.77)	189 (48.09)	N/A^c^
Unique telephone numbers, n	609	593	254	1456
Median number of calls from a single number	1	2	1	2
Maximum number of calls from a single number, n	34	25	24	77

^a^IVR: interactive voice response.

^b^There is an overlap in the callers to IVR and live agents (187 phone numbers) and live agents and voicemail (86 phone numbers), that is, multiple calls from a single number choosing IVR, live agent, or voicemail on different calls. Therefore, the total in the table exceeds the figure (1125 unique telephone numbers) mentioned above. The numbers in the “Overall” column are calculated from the combined call data set of IVR, live agent, and voicemail rather than the summation of the other column figures. Further, the 25 unanswered calls are not included.

^c^N/A: not applicable.

**Figure 1:**
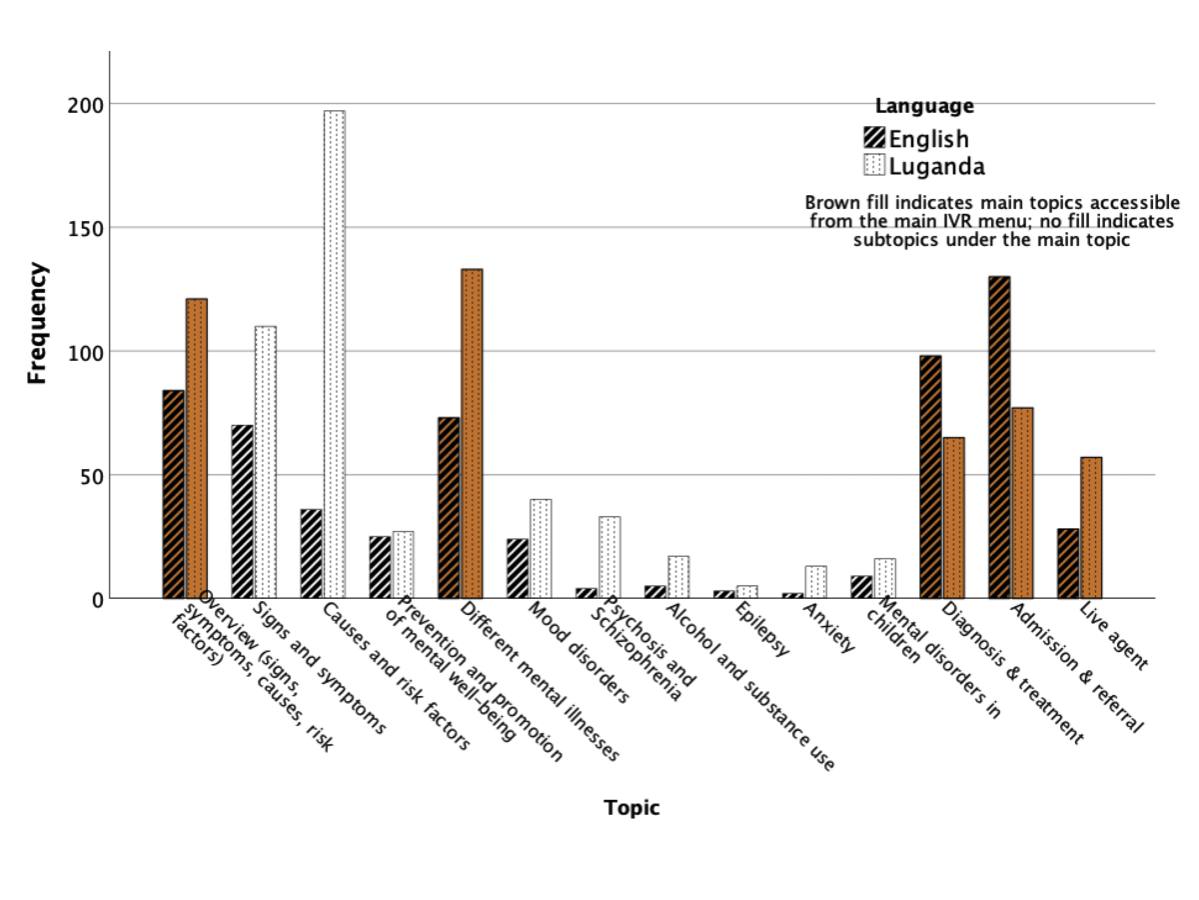
Mental health messages listened to in the IVR. IVR: interactive voice response.

Figure 1 illustrates the mental health information topics accessed through the IVR, along with their respective frequencies (direct calls and replays combined). The most frequently sought information pertained to the general understanding of mental illnesses, including their signs, symptoms, and causes. This accounted for 670 (44.61%) of the 1502 calls for which the IVR topic was recorded in the system. In addition, Luganda was the predominant language for IVR messages (911/1502, 60.65%).

**Figure 2:**
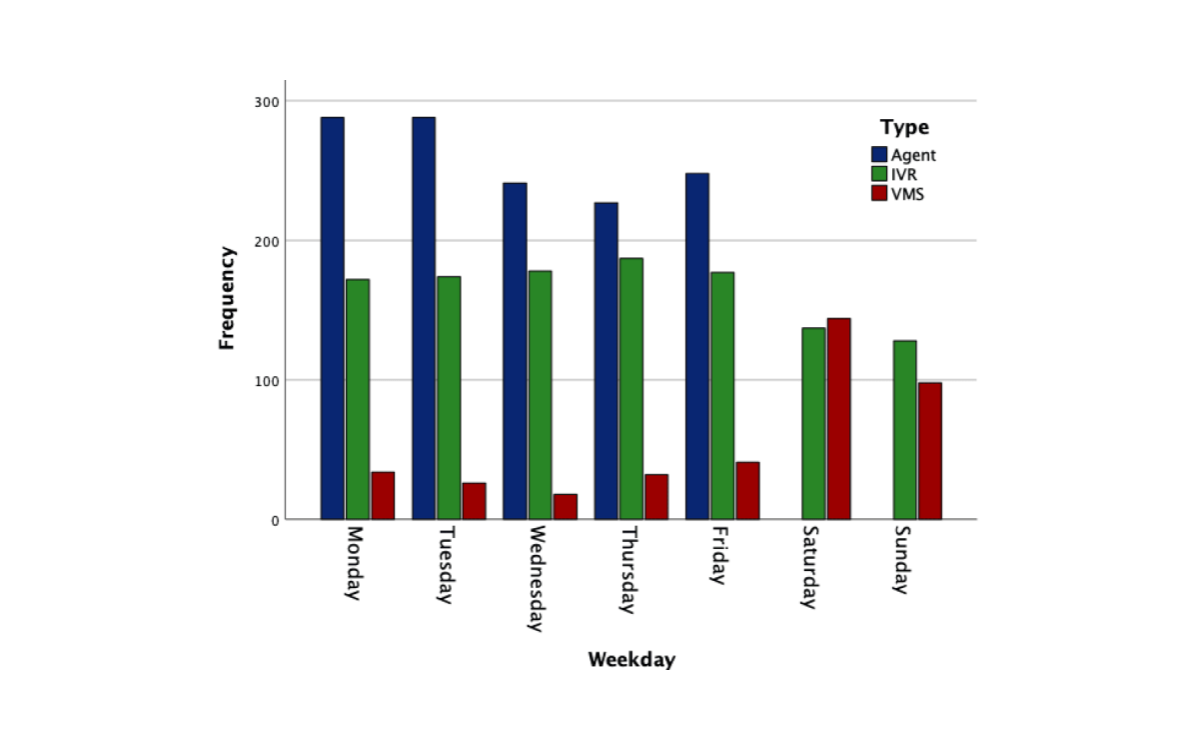
Distribution of call types per day of the week. There were no live agents on Saturdays and Sundays. IVR: interactive voice response; VMS: voicemail system.

Multimedia Appendix 1 presents the call trends over the 18-month period, while Multimedia Appendix 2 displays the distribution of calls throughout the day, and Figure 2 illustrates call distribution across the days of the week. Overall, usage remains consistently sustained over time, with notable spikes in call volume corresponding to marketing and sensitization events, such as community outreach initiatives where the call center number was shared with patients and the general public. Relatively more calls were made earlier in the week and during daytime hours compared with weekends and nighttime. Call agents, including nurses, psychiatric clinical officers, psychologists, and peer support workers, were available only during office hours (8 AM to 6 PM, Monday to Friday). The system was configured not to route any calls to live agents outside of these hours, which explains the absence of calls to live agents and the prevalence of voicemails on Saturdays and Sundays.

### Usability

#### Characteristics of Usability Survey Participants

A total of 252 callers were surveyed, but 12 were excluded from the analysis due to incomplete usability question responses (eg, resulting from poor phone network connections or lack of time) or because they reported difficulty recalling their experience with the call center service. The remaining 240 surveys analyzed included responses from caregivers of patients with mental health issues (n=144, 60.0%), members of the general public (n=46, 19.2%), patients with mental health issues (n=44, 18.3%), health care providers (n=5, 2.1%), and peer support workers (n=1, 0.4%).

The majority of participants were male (n=143, 59.6%), spoke English (n=180, 75.0%), had postsecondary education (n=164, 68.3%), lived within 30 km (approximately 1 hour or less) from Butabika Hospital (n=187, 77.9%), and were aged 25-34 years (n=97, 40.4%) or 35-44 years (n=63, 26.3%). Among the patients (ie, those categorized as either a patient or caregiver; n=188), most had experienced their illness for over a year (n=147, 78.2%) and were already receiving treatment (n=134, 71.3%). The most common mental health issues reported were alcohol and substance-related disorders (n=48, 25.5%) and mood disorders (n=43, 22.9%). Additional participant characteristics are summarized in Table 2.

**Table 2:** Characteristics of usability survey participants (N=240).

Characteristic and categories		Values, n (%)
**Category of participant (caller)**		
	Caregiver	144 (60.0)
	Other^a^	46 (19.2)
	Patients with mental health issues	44 (18.3)
	Health care provider	5 (2.1)
	Peer support worker	1 (0.4)
**Language**		
	English	180 (75.0)
	Luganda	60 (25.0)
**Sex of participant (caller)**		
	Male	143 (59.6)
	Female	97 (40.4)
**Age (years) group of participant (caller)**		
	18-24	39 (16.3)
	25-34	97 (40.4)
	35-44	63 (26.3)
	45 and above	41 (17.1)
**Does the phone belong to you?**		
	Yes	237 (98.8)
	No	3 (1.3)
**Highest level of education completed**		
	University degree or higher	114 (47.5)
	Tertiary but not degree	50 (20.8)
	“A” level	19 (7.9)
	“O” level	33 (13.8)
	Primary school	22 (9.2)
	None	2 (0.8)
**Marital status**		
	Married/cohabiting	117 (48.8)
	Never married	107 (44.6)
	Separated/widowed	16 (6.7)
**Distance to Butabika (30 km is approximately 1 hour travel time)**		
	≤30 km	187 (77.9)
	>30 km	53 (22.1)
**Sex of the patient** ^b^ **(N=188)**		
	Male	108 (57.4)
	Female	80 (42.6)
**Age group (years) of the patient** ^b^ **(N=188)**		
	Under 18	33 (17.6)
	18-24	33 (17.6)
	25-34	68 (36.2)
	35-44	33 (17.6)
	≥45	21 (11.2)
**Complaints** ^c^ **(N=188)**		
	Alcohol and substance-related disorders	48 (25.5)
	Mood disorder (eg, depression, bipolar)	43 (22.9)
	Psychotic disorders	26 (13.8)
	Epilepsy	21 (11.2)
	General enquiry or problem not clear	20 (10.6)
	Physical symptoms/illness	11 (5.9)
	Cognitive problems	9 (4.8)
	Anxiety disorders	5 (2.7)
	Developmental disorders	3 (1.6)
	Sexual and gender identity disorders	2 (1.1)
**When did the illness start? (N=188)**		
	A year or more ago	147 (78.2)
	Less than a year ago	41 (21.8)
**Is the patient on treatment? (N=188)**		
	Yes	134 (71.3)
	Alternative medicine	3 (1.6)
	No	51 (27.1)

^a^Other, for example, included community members reporting a person in the community who seems to have a mental illness and requesting guidance on how to deal with them, news agencies, and students looking for internships.

^b^Callers who said they were caregivers of a patient with mental health issues were asked for details about their patient. Together with callers who were patients themselves, these formed the total number of patients.

^c^Diagnoses reported by the participant; otherwise the research team derived the most likely diagnosis from the reported symptoms.

#### Usability Scores

The Cronbach α for the overall scale was 0.89, while for the subscales it ranged from 0.60 (Ease of Use) to 0.76 (Interaction Quality). Deleting any item resulted in a decrease in the α value.

The overall mean usability score for the system was 4.12 (SD 0.69) on a 5-point scale, which was significantly higher than the midpoint score of 3 (*P*<.001) and also significantly exceeded the recommended target usability score of 4 (*P*=.006) [31,32].

The mean scores for the individual TUQ items ranged from 3.50 (SD 1.28) for the item “The system is able to do everything I would want it to be able to do” to 4.61 (SD 0.80) for the item “I would use the mental health call center again.” All items had scores higher than the midpoint of 3 (all *P*s<.001), and 14 of the 20 items exceeded the recommended score of 4 on a 5-point scale [31,32]. The 6 items that scored below 4 included all 3 items in the Reliability component, as well as 1 item from each of the Usefulness, Interface Quality, and Satisfaction components (Table 3).

**Table 3:** Usability components, questionnaire items, and their means, SDs, and Cronbach α for the subscales (usability attributes).^a^

Usability component and items		Item mean ( 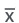 )	SD	*P* value^b^	Mean difference ( 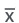 –4)	Component mean^c^		SD		( 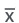 –4)		*P* value		Component α^c^	
**Usefulness**						3.98		0.91		–0.02		.35		.61	
	U1: The mental health call center service improves my access to health care services	4.09	1.11	.11	0.09										
	U2: The mental health call center service saves me time traveling to a hospital or specialist clinic	4.04	1.28	.32	0.04										
	*U3: The mental health call center service provides for my health care needs*	*3.83*	*1.22*	*.02*	–*0.17*										
**Ease of use**						4.41		0.80		0.41		<.001		.60	
	E1: It was simple to use the call center system	4.43	0.89	<.001	0.44										
	E2: It was easy to learn to use the call center system	4.42	0.94	<.001	0.42										
**Interface quality**						4.18		0.75		0.18		<.001		.66	
	UI1: The way I interact with the providers over the call center system is pleasant	4.54	0.91	<.001	0.54										
	UI2: I like using the call center service	4.26	1.06	<.001	0.26										
	UI3: The information is simple and easy to understand	4.47	0.86	<.001	0.47										
	*UI4: This system is able to do everything I would want it to be able to do*	*3.50*	*1.28*	*<.001*	–*0.50*										
**Interaction quality**						4.30		0.81		0.30		<.001		.76	
	UX1: I could easily talk to the mental health provider using the call center service	4.19	1.18	.008	0.19										
	UX2: I could hear the health provider clearly using the call center system	4.54	0.91	<.001	0.54										
	UX3: I felt I was able to express myself effectively	4.43	0.96	<.001	0.43										
	UX4: Using the call center service, I could interact with the health care provider or peer support worker as well as if we met in person	4.07	1.20	.19	0.07										
**Reliability**						3.66		1.03		–0.34		<.001		.60	
	*R1: I think the care provided over the call center service is the same as in-person visits*	*3.54*	*1.32*	*<.001*	–*0.46*										
	*R2: Whenever I made a mistake using the system, I could recover easily and quickly*	*3.83*	*1.24*	*.03*	–*0.17*										
	*R3: The system gave error messages that clearly told me how to fix problems*	*3.68*	*1.40*	*.001*	–*0.32*										
**Satisfaction and future use**						4.17		0.80		0.17		<.001		.66	
	S1: I feel comfortable communicating with the mental health care provider using the call center service	4.24	1.15	<.001	0.24										
	*S2: The call center service is an acceptable way to receive health care services (compared for example to face-to-face)*	*3.68*	*1.31*	*<.001*	–*0.32*										
	S3: I would use mental health call center service again	4.61	0.80	<.001	0.61										
	S4: Overall, I am satisfied with this mental health call center service	4.18	1.11	.007	0.18										
Overall							4.12		0.69		0.12		.006		.89

^a^Items in italics are less than 4, whereas the rest are higher than 4 or equivalent (within its CI).

^b^*P* value: 1-tailed *t* test (whether the mean is significantly less than 4).

^c^Component mean and α were calculated for items in the individual subscale.

The mean scores for the usability components (subscales) ranged from 3.66 (SD 1.03) for Reliability to 4.41 (SD 0.80) for Ease of Use. All scores were significantly higher than the midpoint of 3 (*P*<.001), and all components, except for Reliability, exceeded 4 or fell within their respective CIs.

The overall usability was slightly higher for Luganda speakers (4.24/5) compared with English speakers (4.07/5), but not statistically significant (*P*=.05). However, Luganda speakers demonstrated significantly higher scores in several usability components: interface quality (4.36/5 vs 4.12/5, *P*=.01), interaction quality (4.47/5 vs 4.25/5, *P*=.04), reliability (3.99/5 vs 3.55/5, *P*=.002), and satisfaction and future use (4.39/5 vs 4.10/5, *P*=.008).

There were no statistically significant differences in usability scores based on gender (*P*=.63), among participants living within 30 km (1 hour) from Butabika Hospital compared with those living farther away (*P*=.72), for patients who had experienced mental illness for a year or less versus those with a longer duration (*P*=.60), or for those receiving treatment versus those who were not (*P*=.65). Additionally, no differences in usability scores were observed across various participant categories (*P*=.67), age groups (*P*=.46), levels of education (*P*=.51), or marital status (*P*=.51).

#### Qualitative Comments

A total of 155 comments were coded into 6 themes (Table 4). The majority of the comments (n=60) expressed *appreciation* for the call center initiative, highlighting positive experiences with the service. One participant remarked that she was “very pleasantly surprised that [the hospital has] a toll-free line and it actually worked”. Conversely, 26 comments addressed *usability challenges*, including poor network connectivity, long telephone queues, callback delays, and difficulties navigating the IVR instructions for non–tech-savvy callers.

**Table 4:** Qualitative comments from the usability survey (N=155).

Theme	Description	Frequency, n (%)^a^	Illustrative quotes
Appreciation	Comments where participants expressed appreciation, approval, compliments, or other positive opinions (eg, because it is convenient, free, or improves access to mental health services).	60 (38.7)	interactive and caring people the customer care is good happy [to get] the right information about the prices of the private services before coming to the hospital very pleasantly surprised that [the hospital has] a toll-free line and it actually worked Just thanking you for the service, because otherwise as a person in a rural area far away [I]was stuck with [my] grand daughter and had no other means of getting help. [I] was attend to well. Having access to this information free of charge is wonderful using the toll free number. I t’s a good innovation, it’s good to get the information you need before coming to the hospital
Usability challenges	Comments where participants reported difficulty using the system (eg, due to poor network; long telephone queues before connecting to an agent; difficulty in following the interactive voice response instructions; and mismatch in expectations, such as expecting an agent 24/7 instead of voicemail over the weekend or trying to book an appointment that was not possible).	26 (16.8)	For a village person [someone who is not tech-savvy]the system is difficult There should be clear network and quite environment. T he length of time it takes to get to talk to a responder is too long, it should be the first option and not the last G et more call responders so that a caller does not wait for long on line T he call center responder[s] take long to return voicemail call.
Recommendations for improving the call center	Suggestions for improving the reach and impact of the call center service (eg, more marketing and sensitization of the public about it, adding a video call option, more training for the agents, adding more agents to ensure faster response and 24/7 availability, including specialists to give therapy on phone, adding more languages and agents who understand cultural and religious perspectives of calls, and improve continuity by integrating with other information systems).	48 (31.0)	If the system could be operating 24/7 with a respondent and not just voice mail on weekend and after 8pm. They should keep updating the call center responders to the current information in different departments in the hospital. The call center should have all information or file data concerning the patients so that if one calls in to find out about their patient, there is an answer for them Do more of the sensitization of the number to the public. There should be professional personnel like doctors to answer some technical questions. It would be good if there is a person who can give information depending on the cultural, religious and other affiliation The stuff responding to the callers should have phone etiquette
Recommendations for improving mental health care services in general	Comments or suggestions for improving mental health care services beyond the call center (eg, reducing medicine stock-outs, availing ambulances and pick-up services for patients who might be difficult to handle due to their mental illness, improving patient security and care while they are in Butabika Hospital, having more mental health care providers in rural areas, and other digital services that supplement the call center).	26 (16.8)	We are not getting enough drugs when we come for review yet the drugs are very expensive and patients keep relapsing whenever they miss. ...need to put a psychologist in different centers for easy access to services There should be proper directions when someone is in the hospital Security [should] be tightened so that patients do not escape easily An emergency team should be put in place to pick-up people that have lost their sanity from the community On the Butabika website prices of all services offered at the hospital should be shown to the public clearly for one to know what they need off hand Avail download of prescriptions
Questions about mental illnesses	Participants asked questions related to mental illnesses	6 (3.9)	Is depression curable? What [is the] time span for someone to heal from mental illness?
Issues related to the survey	Comments related to the telephone survey or the items in the questionnaire	4 (2.6)	Send the questionnaire via email Instead of asking someone: “I like using the call center” use “I prefer using the call center [compared to...

^a^There were a total of 155 comments, but some comments were coded into more than 1 theme. Hence, the sum of the frequencies is 170.

Several comments included recommendations for improvement. The majority (48 comments) focused on *enhancing the call center service.* Suggestions included increasing marketing and sensitization efforts to improve outreach, adding more staff to reduce call waiting times and ensure 24/7 availability, providing training for agents, incorporating clinical specialists to offer therapy over the phone, and adding video calling options. Other recommendations focused on *improvements in mental health care services in general.* Suggestions included improving the availability of medications, enhancing patient care and security at Butabika Hospital, increasing access to mental health services in rural areas, and establishing community outreach and ambulance services to assist in transporting patients with unstable mental health conditions. A few comments were related to the usability survey (2 comments) or posed questions about mental illness (6 comments).

### Assessment of Call Conversations by Health Workers

Call conversation ratings are presented in Table 5. The overall mean quality of the call conversations, as assessed by health workers, was 4.35 (SD 0.69) out of 5. Among the raters, rater 3 provided the lowest rating of 3.84 (SD 1.08), while rater 2 assigned the highest score of 4.66 (SD 0.75). The individual quality aspects varied, with scores ranging from 4.27 (SD 0.82) for ending to 4.49 (SD 0.57) for listening.

**Table 5:** Call conversation assessment by health workers.

Quality aspect and rater		Mean (SD)
**All (average)**		
	Rater 1	4.55 (0.75)
	Rater 2	4.66 (0.69)
	Rater 3	3.84 (1.08)
	All raters	4.35 (0.69)
**Rapport**		
	Rater 1	4.69 (0.68)
	Rater 2	4.64 (0.74)
	Rater 3	3.55 (1.39)
	All raters	4.29 (0.72)
**Listening**		
	Rater 1	4.70 (0.65)
	Rater 2	4.73 (0.67)
	Rater 3	4.04 (1.16)
	All raters	4.49 (0.57)
**Analyzing**		
	Rater 1	4.40 (0.99)
	Rater 2	4.64 (0.74)
	Rater 3	3.98 (1.20)
	All raters	4.34 (0.77)
**Motivating**		
	Rater 1	4.45 (0.97)
	Rater 2	4.66 (0.76)
	Rater 3	3.94 (1.27)
	All raters	4.35 (0.80)
**Ending**		
	Rater 1	4.51 (0.90)
	Rater 2	4.61 (0.83)
	Rater 3	3.69 (1.29)
	All raters	4.27 (0.82)

The quality aspects of the call conversations were highly correlated, with correlation coefficients ranging from 0.72 (between rapport and ending) to 0.92 (between analyzing and motivating). Additionally, the overall reliability of the quality rating scale was excellent, with a Cronbach α of 0.97.

The overall call quality showed a significant correlation with overall usability (Pearson *r*=0.34, *P*=.02). The correlations between individual quality aspects and usability components are presented in Multimedia Appendix 3, with the strongest correlation observed between ending and satisfaction (Pearson *r*=0.55, *P*<.001).

There was no association between the type of agent (peer support worker vs professional health care worker) or the nature of the call issue (whether solvable over the phone or not) and the usability scores provided by the callers.

## Discussion

### Principal Findings

This study evaluated the usage and usability of a mental health telephone advice service in Uganda. The findings indicate that all 3 access channels of the mental health telephone service (IVR, live calls, and voicemail) were utilized, and usage remained consistent throughout the evaluation period. The substantial proportion of callers (1153/2863 calls, 40.27%) who stopped at the IVR, despite having the option to speak with a live agent, is encouraging as it suggests a level of acceptability for the IVR system, potentially even a preference for it over direct interaction with an agent. Utilizing IVR alleviates the burden on limited human resources and facilitates access to information outside of regular office hours. The call distribution data (see Multimedia Appendix 2 and Figure 2) illustrate that a significant portion of calls occurred on weekends and during the evening and night when staff were unavailable to respond, highlighting the IVR’s utility in meeting callers’ needs during these times. Additionally, all callers who opted to speak with an agent first navigated through the IVR, thereby accessing automated information before their conversation. In the formative research for this service [24], participants expressed a desire for anonymity to mitigate stigma, and they felt that the call center service provided this necessary confidentiality. Similarly, in another study focused on a cancer awareness service in Uganda [33], callers noted that a key advantage of IVR was the ability to listen to information multiple times, a feature that is often impractical or uncomfortable when interacting with a live agent (eg, when repeatedly requesting the same information). These perspectives may help clarify why a significant number of callers opted not to speak with an agent. Conversely, some survey participants indicated a preference for having the option to speak to an agent presented as the first choice. This suggests that the higher percentage of IVR users could be attributed to our system design, which intentionally prioritized IVR options over direct access to an agent, rather than reflecting a definitive user preference.

It is noteworthy that only a small proportion of the callers (44/240, 18.3%) were patients themselves, while the majority were caregivers (144/240, 60.0%) or other members of the general public (46/240, 19.2%). Butabika Hospital serves as a national referral center for individuals with severe mental health disorders, and prior studies in Uganda have indicated that people typically seek care only when mental health issues reach a severe level [34,35]. Such patients may struggle to arrange their own care or comprehend information provided through telephone services. Additionally, individuals with mental disorders often experience social isolation and may not have access to phones, further limiting their ability to seek assistance [24,36]. The significant role of caregivers (family members) must be taken into account when designing mental health interventions. For telephone services like ours, it is crucial to thoroughly explore and address the information needs of caregivers, as these may differ from those of the patients themselves. Understanding and accommodating these unique needs can enhance the effectiveness of mental health services and improve support for both patients and their caregivers [36].

The results indicated significantly high scores across various usability aspects and for the overall system, surpassing the recommended target usability scores [31,32]. This can be attributed to the user-centered approach applied during the design and implementation of the system. By prioritizing the unique circumstances of the target users in Uganda—such as their literacy levels, information needs, mental health care experiences, cultural norms, health-seeking behaviors, and familiarity with technology—we were able to effectively address their specific requirements and enhance the system’s usability. Similar findings of high usability—particularly regarding satisfaction, intention for future use, ease of use, and perceived usefulness—have been reported in other studies on tele-mental health care in the United States [37], South Korea [38], the United Kingdom [39], and Italy [40], albeit within the context of COVID-19 lockdowns. This indicates that cultural factors, such as the “power distance” [41,42] between health care workers and patients—which tends to be higher in Africa [43-45]—did not significantly impact usability scores. The lack of fear among participants in rating the service may suggest a shift in attitudes toward the provider-patient relationship, which is often characterized by paternalism in African contexts. We could not find any studies in Uganda that have assessed aspects of the usability of digital health solutions from the client’s perspective. As triangulated from the qualitative comments and call quality ratings provided by health care providers in our study, participants appreciated the convenience and the ability to access information through the telephone service at no cost. Additionally, it was evident that some participants had previously encountered negative experiences with other call center services or did not expect this hospital to establish one that “actually worked” and featured “pleasant and caring” agents. These factors contributed to the high usability scores observed in our study.

There was no difference in usage (number of calls) and efficiency (duration of calls) overall between English and Luganda. However, a slight majority of calls that stopped at the IVR were in Luganda. Additionally, the IVR options (ie, mental health information topics) sought differed for the 2 languages. Luganda-speaking callers primarily sought information to understand mental illnesses, including what mental illnesses are and what causes them. By contrast, English-speaking callers were more interested in the management of mental illnesses, specifically information on diagnosis, treatment, and the referral process. English proficiency serves as a proxy for formal literacy in Uganda, as English is taught in schools and used in health care and other official communications, except for clients with low literacy. The finding that callers with less literacy required more information for a basic understanding of mental illnesses is not surprising. Literate community members can access various mental health information sources, including online resources and print media, which may lead to fewer unmet mental health information needs among them. This is further supported by the finding that usability scores were slightly higher for Luganda speakers than for English speakers, suggesting that they appreciate the service more or consider it more useful, as it represents one of the few sources of mental health information available to them. As noted by Park et al [38], when users lack alternatives—whether due to COVID-19 lockdowns or, in Uganda’s case, limited access to mental health care services in general—they may rate the telephone service more optimistically.

Similar to our findings, other studies have reported that the reliability component of usability is often ranked the lowest, with users expressing a desire for physical encounters with mental health care providers [37,39], despite their appreciation of the telephone service. Nonverbal communication plays a crucial role in mental health, as it contributes to comprehensive assessment and the development of a therapeutic alliance. Therefore, telephone services should supplement rather than replace physical clinic visits. Videoconferencing options have also been proposed in our study and in others [37,39]; however, this could be constrained in Uganda due to limited access to the internet and smart devices in rural areas.

Connolly and colleagues [13] state that the evidence base for the usability of mental health apps is still uncertain and argue that simple telephone features can effectively deliver mental health services instead of relying on expensive smart apps with poor usability. Our study contributes to the literature on usability and demonstrates how basic phone features, such as IVR and voice calls, can provide significant value and a positive user experience. We also demonstrated efficient use of human resources, as we did not hire new staff but instead utilized over 130 hours from existing staff and volunteer peer support workers. With very few unanswered calls (25/1710 calls, 1.46%) and no reports from callers of congested telephone lines or long queues, we could potentially gain more hours from staff if we market the service more effectively to increase demand. Analyzing usage trends can help identify the busiest days of the week or hours of the day, thereby informing staff scheduling, further improving efficiency, and reducing the number of unanswered calls even when demand rises.

### Limitations

There are a few limitations to this study. First, the usability survey was not conducted in real time; instead, it occurred a few days or even weeks after the participant called the mental health telephone service. This delay could have introduced recall bias, as some participants reported difficulty remembering their experience, leading to the termination of their surveys. However, it is also possible that some participants continued with the survey despite their poor recall. Future studies should aim to collect user feedback immediately after the call to enhance the accuracy of the responses. Moreover, the possibility of selection bias exists, where individuals who had a positive experience with the service may have been more likely to agree to participate in the survey, while those who encountered network connectivity challenges or had negative experiences may have been less reachable or less willing to engage. This potential bias could affect the overall assessment of usability and user satisfaction.

Another limitation is the cross-sectional nature of the survey. Users of the mental health telephone service called the system a median of 2 times, and their experiences may have varied across these encounters. Collecting feedback immediately after each call and tracking responses from each caller during different encounters could provide more insights into factors influencing usability, such as the specific agent who answered the call. Qualitative responses indicated that some participants felt the communication skills of certain agents were lacking; however, we could not identify the agents to subanalyze the usability scores associated with each individual.

Lastly, this study focused on a single system at a single site, which may limit the generalizability of the findings. Without a comparative system, interpreting individual usability scores could be challenging. Typically, usability evaluations are conducted using an A/B setup to compare different systems or versions of the same system. In our study, however, we utilized the recommended cutoffs derived from hundreds of usability studies as reference means for our *t* tests, rather than relying solely on the midpoint of the Likert scale [31,32].

### Study Implications

First, the implications of our findings for policy and practice suggest that digital health tools are both acceptable and useful for addressing gaps in mental health care delivery in low- and middle-income countries. Therefore, these tools, particularly those utilizing simple and widely accessible mobile phones, should be embraced as a viable solution for improving mental health services. Second, using user-centered design and participatory methods is essential to ensure good usability in digital health services. Additionally, assessing both usage and usability is crucial to enhance the scientific evidence on mHealth. This includes understanding the information consumed by users, preferred communication channels, types of users, and any challenges they face. Such assessments will inform targeted improvements in digital health services, ultimately leading to better user experiences and outcomes.

### Conclusion

This study found sustained usage of a mental health telephone service in Uganda, with positive user experiences, high satisfaction, and strong intentions for future use across diverse user groups. mHealth solutions like ours should be embraced and replicated to enhance the delivery of mental health services and address other disease areas in Uganda and similar low-resource settings. However, attention should be paid to user experience, and the design and implementation of the system (eg, content and access channels) should align with the preferences and characteristics of different users to ensure good usability and sustained use.

## Appendix

Multimedia Appendix 1 Call trends over time.

Multimedia Appendix 2 Calls trends across the time of the day.

Multimedia Appendix 3 Correlations between call conversation aspects and usability components.

## Abbreviations

IVR: interactive voice response
mHealth: mobile health
STARE-HI: Statement on Reporting of Evaluation Studies in Health Informatics
TUQ: Telehealth Usability Questionnaire

## Data Availability

The data sets generated during or analyzed in this study are not publicly available because they contain personal data (eg, telephone numbers), but are available from the corresponding author on reasonable request.
