# Supplementary figures and images for: Assessing the Usage and Usability of a Mental Health Advice Telephone Service in Uganda: Mixed Methods Study
Source: J Med Internet Res. 2024 Oct 21;26:e65692. doi: 10.2196/65692 (PMC11535793; doi:10.2196/65692)

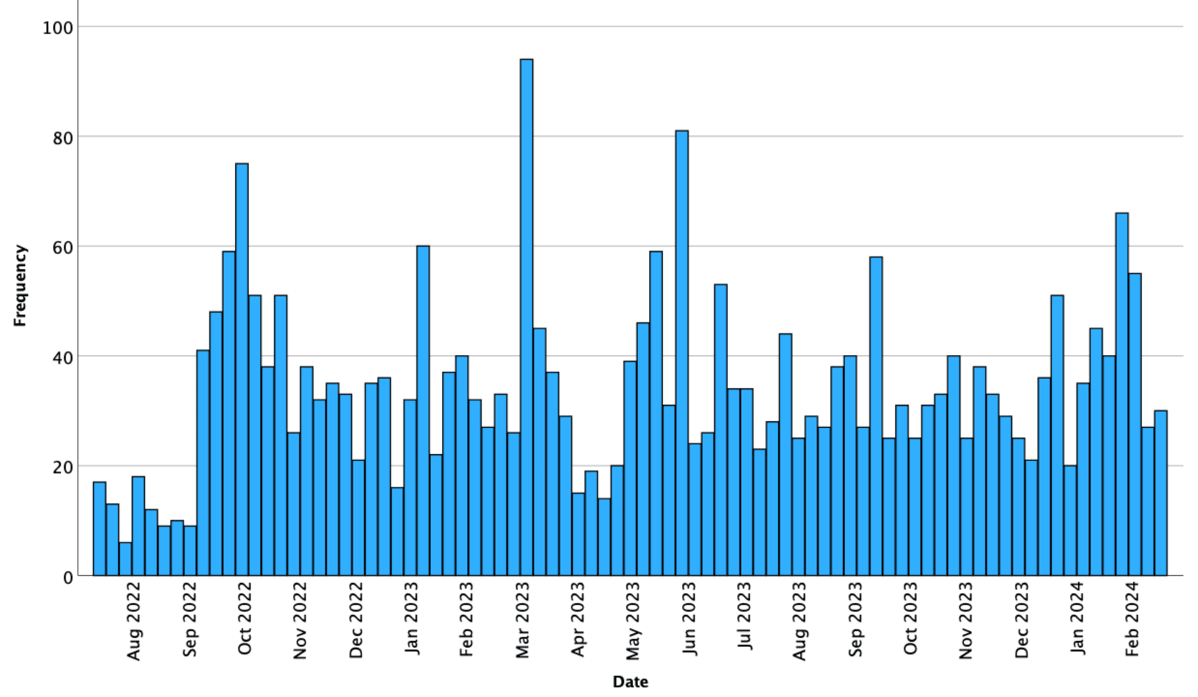

Supplement: Multimedia Appendix 1 [file jmir_v26i1e65692_app1.png]

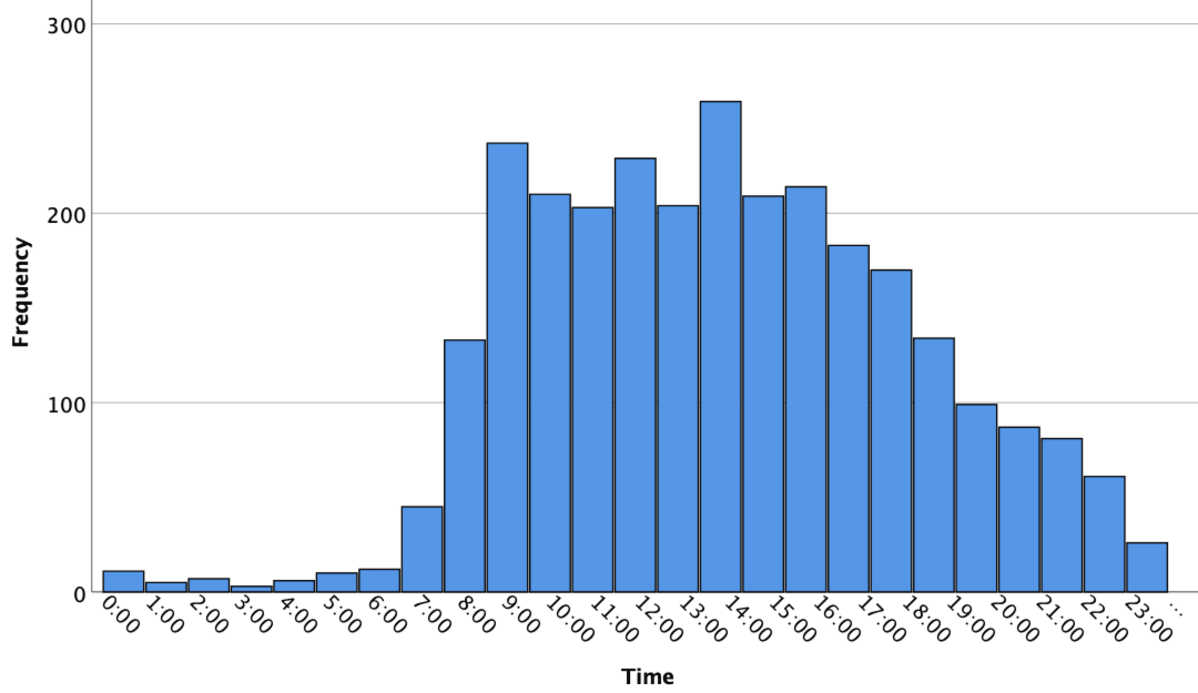

Supplement: Multimedia Appendix 2 [file jmir_v26i1e65692_app2.png]
