# Supplementary material for: Assessing the Usage and Usability of a Mental Health Advice Telephone Service in Uganda: Mixed Methods Study
Source: J Med Internet Res. 2024 Oct 21;26:e65692. doi: 10.2196/65692 (PMC11535793; doi:10.2196/65692)
Supplement: Multimedia Appendix 3 [file jmir_v26i1e65692_app3.docx]

**Multimedia Appendix 3.** Correlations between call conversation aspects and usability components.

| Correlation | Rapport | Listening | Analyzing | Motivating | Ending | Conversation quality (overall) | Usability (overall) | Usefulness | Satisfaction | Ease of use | Interface quality | Interaction quality | Reliability |
| --- | --- | --- | --- | --- | --- | --- | --- | --- | --- | --- | --- | --- | --- |
| Rapport | 1.00 | 0.851^a^ | 0.848^a^ | 0.835^a^ | 0.723^a^ | 0.904^a^ | 00.23 | 0.306^b^ | 0.287^b^ | –0.03 | 0.17 | 0.402^a^ | 0.18 |
| Listening | 0.851^a^ | 1.00 | 0.885^a^ | 0.891^a^ | 0.823^a^ | 0.943^a^ | 0.26 | 0.312^b^ | 0.343^b^ | –0.11 | 0.23 | 0.325^b^ | 0.24 |
| Analyzing | 0.848^a^ | 0.885^a^ | 1.00 | 0.917^a^ | 0.825^a^ | 0.956^a^ | 0.315^b^ | 0.354^b^ | 0.361^b^ | 0.01 | 0.27 | 0.341^b^ | 0.28 |
| Motivating | 0.835^a^ | 0.891^a^ | 0.917^a^ | 1.00 | 0.876^a^ | 0.967^a^ | 0.27 | 0.369^b^ | 0.345^b^ | –0.10 | 0.27 | 0.294^b^ | 0.23 |
| Ending | 0.723^a^ | 0.823^a^ | 0.825^a^ | 0.876^a^ | 1.00 | 0.913^a^ | 0.479^a^ | 0.382^a^ | 0.550^a^ | 0.11 | 0.479^a^ | 0.359^b^ | 0.403^a^ |
| Conversation quality (overall) | 0.904^a^ | 0.943^a^ | 0.956^a^ | 0.967^a^ | 0.913^a^ | 1.00 | 0.337^b^ | 0.368^b^ | 0.407^a^ | –0.02 | 0.311^b^ | 0.364^b^ | 0.289^b^ |
| Usability (overall) | 8.00 | 0.26 | 0.315^b^ | 0.27 | 0.479^a^ | 0.337^b^ | 1.00 | 0.858^a^ | 0.920^a^ | 0.787^a^ | 0.929^a^ | 0.881^a^ | 0.733^a^ |
| Usefulness | 0.306^b^ | 0.312^b^ | 0.354^b^ | 0.369^b^ | 0.382^a^ | 0.368^b^ | 0.858^a^ | 1.00 | 0.717^a^ | 0.534^a^ | 0.759^a^ | 0.669^a^ | 0.404^a^ |
| Satisfaction | 0.287^b^ | 0.343^b^ | 0.361^b^ | 0.345^b^ | 0.550^a^ | 0.407^a^ | 0.920^a^ | 0.717^a^ | 1.00 | 0.596^a^ | 0.867^a^ | 0.737^a^ | 0.614^a^ |
| Ease of use | –0.03 | –0.11 | 0.01 | –0.10 | 0.11 | –0.02 | 0.787^a^ | 0.534^a^ | 0.596^a^ | 1.00 | 0.740^a^ | 0.601^a^ | 0.523^a^ |
| Interface quality | 0.17 | 0.23 | 0.27 | 0.27 | 0.479^a^ | 0.311^b^ | 0.929^a^ | 0.759^a^ | 0.867^a^ | 0.740^a^ | 1.00 | 0.677^a^ | 0.609^a^ |
| Interaction quality | 0.402^a^ | 0.325^b^ | 0.341^b^ | 0.294^b^ | 0.359^b^ | 0.364^b^ | 0.881^a^ | 0.669^a^ | 0.737^a^ | 0.601^a^ | 0.677^a^ | 1.00 | 0.544^a^ |
| Reliability | 0.18 | 0.24 | 0.28 | 0.23 | 0.403^a^ | 0.289^b^ | 0.733^a^ | 0.404^a^ | 0.614^a^ | 0.523^a^ | 0.609^a^ | 0.544^a^ | 1.00 |

^a^Correlation is significant at the .01 level (2-tailed).

^b^Correlation is significant at the .05 level (2-tailed).
